# Suppression of Cholangiocarcinoma Cell Growth by Human Umbilical Cord Mesenchymal Stem Cells: A Possible Role of Wnt and Akt Signaling

**DOI:** 10.1371/journal.pone.0062844

**Published:** 2013-04-30

**Authors:** Juan Liu, Guoqing Han, Hui Liu, Chengyong Qin

**Affiliations:** Department of Gastroenterology, Provincial Hospital Affiliated to Shandong University, Jinan, China; University of Kentucky, United States of America

## Abstract

Emerging evidence indicates that human mesenchymal stem cells (hMSCs) can be recruited to tumor sites, and affect the growth of human malignancies. However, little is known about the underlying molecular mechanisms. Here, we observed the effects of hMSCs on the human cholangiocarcinoma cell line, HCCC-9810, using an animal transplantation model, and conditioned media from human umbilical cord-derived mesenchymal stem cells (hUC-MSCs). Animal studies showed that hUC-MSCs can inhibit the growth of cholangiocarcinoma xenograft tumors. In cell culture, conditioned media from hUC-MSCs inhibited proliferation and induced apoptosis of tumor cells in a dose- and time-dependent manner. The proliferation inhibition rate increased from 6.21% to 49.86%, whereas the apoptosis rate increased from 9.3% to 48.1% when HCCC-9810 cells were cultured with 50% hUC-MSC conditioned media for 24 h. Immunoblot analysis showed that the expression of phosphor-PDK1 (Ser241), phosphor-Akt (Ser 437 and Thr308), phosphorylated glycogen synthase kinase 3β (phospho-GSK-3β^Ser9^), β-catenin, cyclin-D1, and c-myc were down-regulated. We further demonstrated that CHIR99021, a GSK-3β inhibitor reversed the suppressive effects of hUC-MSCs on HCCC-9810 cells and increased the expression of β-catenin. The GSK-3β activator, sodium nitroprusside dehydrate (SNP), augmented the anti-tumor effects of hUC-MSCs and decreased the expression of β-catenin. IGF-1 acted as an Akt activator, and also reversed the suppressive effects of hUC-MSCs on HCCC-9810 cells. All these results suggest that hUC-MSCs could inhibit the malignant phenotype of HCCC-9810 human cholangiocarcinoma cell line. The cross-talk role of Wnt/β-catenin and PI3K/Akt signaling pathway, with GSK-3β as the key enzyme bridging these pathways, may contribute to the inhibition of cholangiocarcinoma cells by hUC-MSCs.

## Introduction

Intrahepatic cholangiocarcinoma (ICC) is a malignancy whose pathogenesis involves abnormal biliary epithelial differentiation [1]. The incidence of ICC is increasing worldwide, and it is the second most common form of primary liver cancer next to that of hepatocellular carcinoma. Despite advances in diagnosis and treatment, most patients present with advanced metastatic lesions that are not amenable to surgical extirpation or liver transplantation [2], [3]. Furthermore, current chemotherapy regimens used to treat ICC offer very limited benefit in terms of patient survival.

Mesenchymal stem cells possess a multiple-differentiation potential which permits these cells to differentiate into a variety of mesodermal cell lineages, including bone, cartilage, adipose, tendon and muscle [4]. Therefore, they are considered to contribute to endogenous organ and tissue repair [5]. In contrast to hMSCs from other sources, hUC-MSCs have attracted much attention due to their availability, low immunogenicity, as well as strong tropism for tumors [6]. With regard to the latter property, a number of studies have focused on the relationship between stem cells and tumor cells. The ability of MSCs to migrate to tumors has encouraged investigation of MSCs as therapeutic tools [7], [8]. Stem cell transplantation has been used in the treatment of several hematologic [9] and non-hematologic [10], [11] malignancies. Previous studies have shown that the development and growth of some human solid malignancies can be inhibited by MSC [12]–[14]. Other studies have demonstrated that hMSCs may inhibit tumor cell phenotypes by secreting certain soluble factors [14]–[16]. Because the mechanism of hUC-MSCs effects on human intrahepatic cholangiocarcinoma has not been reported, in the current study, we sought to shed light on this phenomenon.

## Materials and Methods

### Cell Culture

After obtaining the mothers’ written informed consent, UC-MSCs were isolated from the umbilical cords of full-term newborns who were delivered in the Provincial Hospital Affiliated to Shandong University. All experiments were carried out in Central Laboratory, Provincial Hospital Affiliated to Shandong University, with prior approval from the Provincial Hospital Affiliated to Shandong University Medical Institutional Ethical Committee. The mesenchymal stem cell clones were cultured in Dulbecco’s modified Eagle’s medium with low glucose (DMEM, Hyclone, Logan, Utah, USA) supplemented with 10% fetal calf serum (Hyclone). All hMSCs were used in the experiments before reaching the sixth passage. Flow-cytometric analysis of cell surface antigens and differentiation assays were used to identify the hUC-MSCs [17]. Human intrahepatic cholangiocarcinoma cell lines (HCCC-9810), human esophageal carcinoma cell lines (Eca-109), human breast cancer cell lines (MCF-7), human liver cell lines (L-02) and human umbilical vein endothelial cells (HUVECs) were obtained commercially (Keygen Biotech, China) and cultured in RPMI 1640 medium (Hyclone) containing 10% fetal calf serum, 100 U/ml penicillin, 100 mg/ml streptomycin at 37°C in humidified atmosphere containing 5% CO_2_.

### Preparation of Conditioned Media

hUC-MSCs and HUVECs (as a negative control) were cultured to 100% confluence as described above. The conditioned media were filtered through the 0.22 µm pore sterile filter and stored at –80°C until use. In each experiment, HCCC-9810 cells were treated with a mixture of RPMI 1640 medium and conditioned media (in ratios of 9∶1 3∶1, 1∶1, and 1∶3) containing 10% fetal calf serum, and the culture media were replaced every 24 h.

### BALB/c Nude Mice Transplantation

We obtained BALB/c nude mice from the Institute of Zoology, Chinese Academy of Sciences (Beijing, China). All animal experiments were carried out in accordance with a protocol approved by the Shandong University Institutional Animal Care and Use Committee (IACUC). Mice 4–6 weeks old were kept in pathogen-free conditions as described [18], and divided into four groups. Groups 1 and 2 consisted of mice that were treated with a mixture of equal numbers of HCCC-9810 cells (1×10^6^) and hUC-MSCs (1×10^6^), or with a mixture of equal numbers of HCCC-9810 cells (1×10^6^) and HUVECs (1×10^6^). Cells were combined and injected subcutaneously into the nape region of the mice under aseptic conditions as previously described [14]. Mice in Groups 3 and 4 were subcutaneously injected with hUC-MSCs (1×10^6^) only or HCCC-9810 cells (1×10^6^) only, respectively. At 50 days post-injection, the average tumor volumes in these groups were evaluated by measuring the length and width, and mice in the fourth group were further divided into three subgroups. Two groups were treated with the injections of 1 ml 50% conditioned media from hUC-MSCs or HUVECs in the tumor sites every 3 days. Mice in the third group received no treatment. Mice were sacrificed by cervical dislocation, and growth of the tumor was evaluated up to day 70 after cell inoculation.

### Co-cultures of hMSCs and Tumor Cells

Co-culture systems were established by using transwell 6-well plates (0.4 µm pore, polycarbonate membrane; Costar, Cambridge, USA). HCCC-9810 cell suspensions (1 ml, 6×10^5^ cells) were loaded in the upper inserts, and hMSCs cell suspensions (1 ml, 6×10^5^ cells) were put into the lower compartment of the culture well. HUVECs (1 ml, 6×10^5^ cells) were used as a control. Each group had three wells. The numbers of tumor cells in the inserts were counted in triplicate under a microscope after incubation for 48 h, and the results were expressed as means.

### Cell Proliferation Measurement

The cell proliferation was measured using MTT [3-(4,5-dimethyl-thiazol-2-yl)-2,5-diphenyltetrazoliumbromide assay, Sigma] which is based on the conversion of MTT to MTT-form-azan, as previously described [19]. In order to choose the optimum inhibition time, we treated HCCC-9810 cells with conditioned media for 12, 24 and 48 h. The inhibition rate was calculated with the following formula: Inhibition Rate (%) = (1−(OD_Test_/OD_Ctr_))×100%. In order to verify whether the inhibitory effect of MSC conditioned media was specific for HCCC-9810 cells, we also carried out MTT assays on a human esophageal carcinoma cell line Eca-109, human breast cancer tumor line MCF-7, and human liver cell line L-02.

### Colony-forming Assay

HCCC-9810 cells with a final concentration of 10^3^ cells/ml were suspended in RPMI 1640 medium containing 50% conditioned media from hUC-MSCs, and seeded onto plates (60 mm). HCCC-9810 cells treated with 50% conditioned media from HUVECs and RPMI 1640 medium (Mock) were used as a control. Media containing 10% fetal calf serum were replaced every 3 days. After incubating for 2 weeks, colony-forming units were inspected microscopically by using a 40× objective.

### Apoptosis Assay

To verify whether the inhibition of cell proliferation by hUC-MSCs was mediated by apoptosis, chromatin morphology was observed by fluorescence microscopy after DNA staining with 0.5 mg/L Hoechst 33258 (Keygen Biotech, China). The percentage of apoptotic cells was calculated as the number of apoptotic cells compared to the number of total cells counted in randomly selected fields [20]. Images from Hoechst stained samples were acquired by using a Leica ™ fluorescence microscope equipped with a Leica ™ camera. We further chose a DNA ladder assay to detect apoptosis. HCCC-9810 cells were treated with conditioned media for 48 h. Cells were harvested and resuspended in lysis buffer (1 mM EDTA, 10 mM Tris [pH 8.0], 1% SDS, and 1 µg/ml proteinase K). After 1 h incubation at 37°C, RNase A was added and incubation continued for another hour. A crude DNA preparation was extracted twice with phenol/chloroform/isoamyl alcohol (25∶24:1). Cell lysate samples were subsequently run at 100 V on a 1.5% agarose gel containing ethidium bromide (EtBr, Sigma). The gels were examined under ultraviolet light and photographed [20].

### Western Blot Analysis

Expression levels of total-Akt, phospho-Akt, total-GSK-3β, phospho-GSK-3β, β-catenin, cyclin-D1, and c-myc were measured in HCCC-9810 cells by immunoblot analysis. Cells growing in 50% conditioned media for 48 h were harvested. Fractionated nuclear and cytosolic protein lysates were obtained using nuclear extraction buffer [20 mmol/L Tris-HCl (pH 7.5), 420 mmol/L NaCl, 1.5 mmol/L MgCl2, 0.2 mmol/L EDTA, 25% glycerol, 1 mmol/L EDTA, 1 mmol/L DTT, 0.1 mmol/L phenylmethylsulfonyl fluoride (PMSF)] or hypotonic lysis buffer [10 mmol/L HEPES (pH 7.9), 60 mmol/L KCl, 0.3% NP40, 1 mmol/L EDTA, 1 mmol/L DTT, 0.1 mmol/L PMSF]. Total cell protein lysates were extracted from cells in radioimmunoprecipitation assay buffer [25 mmol/L Tris (pH 7.8), 2 mmol/L EDTA, 20% glycerol, 0.1% NP40, 1 mmol/L DTT]. All protein extraction buffers were supplemented with MiniComplete protease inhibitor cocktail (Roche Diagnostic), 1 mmol/L NaF, and 1 mmol/L NaO3V4. Proteins were subjected to SDS-PAGE analysis, transferred to nitrocellulose membranes and incubated with primary antibodies against phospho-PI3K (1∶1000 dilution, Cell Signaling), phospho-PDK1^Ser241^ (1∶1000 dilution, Cell Signaling), Akt (1∶2000 dilution, Cell Signaling), phospho-Akt^Ser473^ (1∶1000 dilution, Cell Signaling), phospho-Akt^Thr308^ (1∶1000 dilution, Cell Signaling), GSK-3β (1∶1 000 dilution, Cell Signaling), phospho-GSK-3β^Ser9^ (1∶1 000 dilution, Cell Signaling), β-catenin (1∶1000 dilution, Abcam), c-Myc (1∶1 000 dilution, Abcam), cyclin-D1 (1∶1000 dilution, Abcam) and β-actin (1∶5000 dilution, Abcam). After incubation with peroxidase-conjugated affinipure goat anti-mouse secondary antibodies (1∶5000 dilution, Abcam), protein bands were detected using an enhanced chemiluminescence reagent (Sigma, USA).

### Indirect Immunofluorescence Staining

Indirect immunofluorescence staining was used to examine the expression of p-Akt, p-GSK-3β, and the localization of β-catenin in HCCC-9810 cells after 48 h of incubation with conditioned media [21]. Cells were fixed at 37°C for 30 min using 4% para-formaldehyde (Sigma) followed by incubation in blocking solution (3% normal goat serum) at room temperature for 30 min. Then, cells were incubated overnight at 37°C in 0.05% triton-X100 (Sigma) containing a mouse monoclonal antibody that specifically recognizes human β-catenin (1∶100 dilution, Abcam), phospho-Akt (1∶50 dilution, Cell Signaling), phospho-GSK-3β (1∶100 dilution, Cell Signaling), followed by 10 washes with phosphate-buffered saline (PBS). As a negative control, cells were incubated in PBS without these antibodies. The cells were incubated with rhodamine (TRITC)-conjugated goat-anti-mouse IgG (1∶50 dilution, Sigma), followed by ten washes with PBS. DAPI was used to stain the nuclei. Images of cells were acquired by using a LeicaTM fluorescence microscope equipped with a LeicaTM camera.

### Chemicals and Viability Assays

CHIR99021 (Stemgent) was dissolved in DMSO (Sigma). Sodium nitroprusside dehydrate (SNP, Sigma) was dissolved in distilled water. HCCC-9810 cells were incubated with the GSK-3β activator SNP, and the GSK-3β inhibitor CHIR99021 as a single treatment or in combination with hUC-MSC conditioned media for 48 h, and the cell survival was measured by using MTT assays [19]. IGF-1 (GenScript) was dissolved in distilled water prior to use. HCCC-9810 cells were treated with increasing concentrations of MSC conditioned media (10%, 25%, 50% and 75%) for 24 h, with or without IGF-1 (200 ng/mL) preincubation for 15 min, and then cell viability was examined by an MTT assay. In all experiments, the final concentration of DMSO did not exceed 0.1%.

### Statistical Analysis

SPSS 16.0 software was used for all statistical analysis. Statistical significance was assessed by comparing mean values (±SD) using the Student’s *t*-test for independent groups.

## Results

### Identification of Human Umbilical Cord Mesenchymal Stem Cells

**Figure 1:**
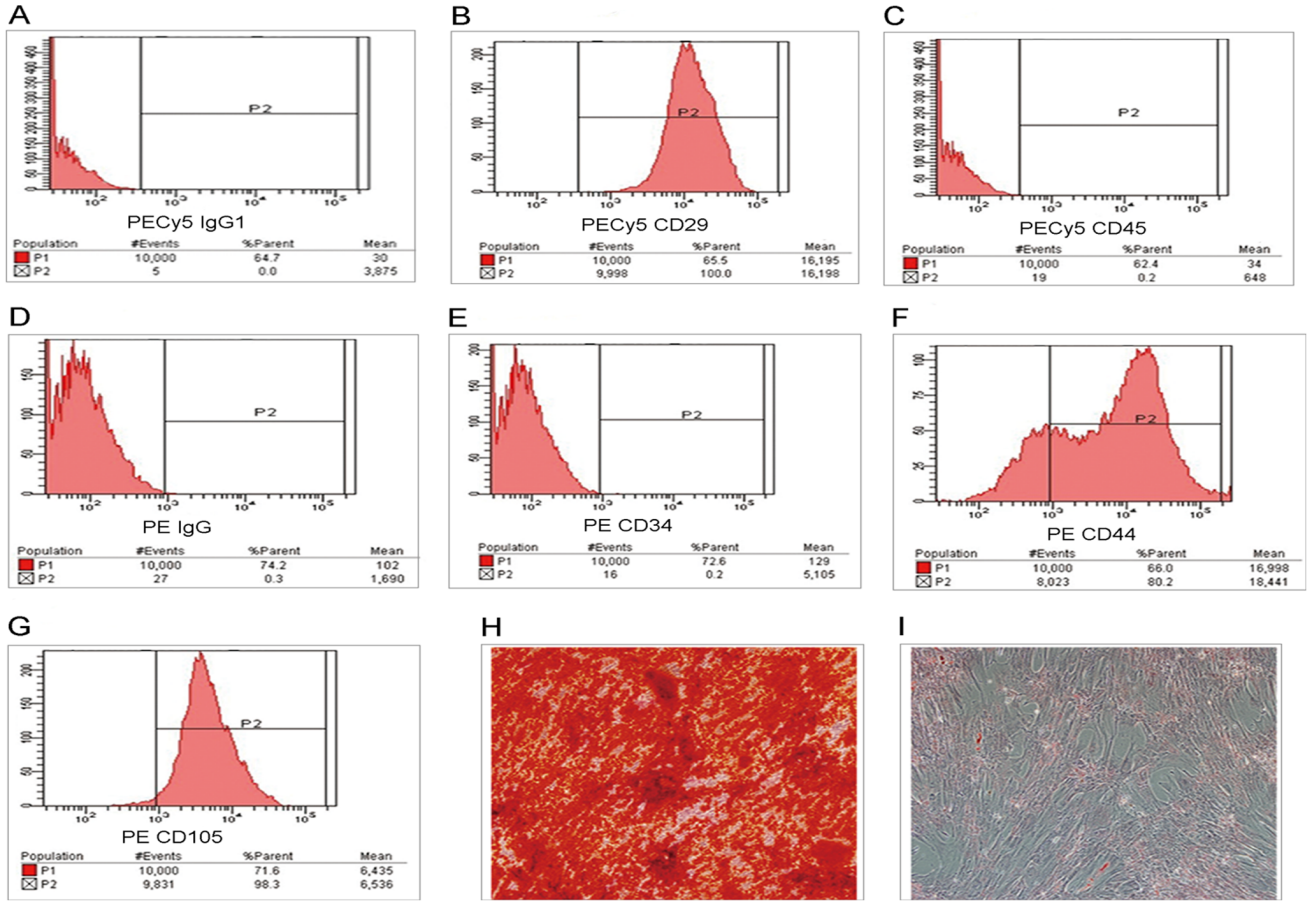
Characterization and differentiation of hUC-MSCs. A–G, Flow-cytometric analysis of cell surface antigens of hUC-MSC; H, Alizarin red S staining of osteogenic differentiated hUC-MSCs (400×); I, Oil red O staining of adipogenic differentiated hUC-MSCs (400×).

The cloned cells from fetal umbilical cords expressed characterized cell surface markers. Flow-cytometric analysis of cell surface antigens showed that the cells were positive for CD44, CD29, and CD105, but were negative for CD34 and CD45 (Fig. 1A–1G). In addition, their ability to differentiate into multiple cell lineages, including bone and fat was tested and verified (Fig. 1H and 1I).

### Intrahepatic Cholangiocarcinoma Cell Proliferation was Inhibited by hUC-MSCs

**Figure 2:**
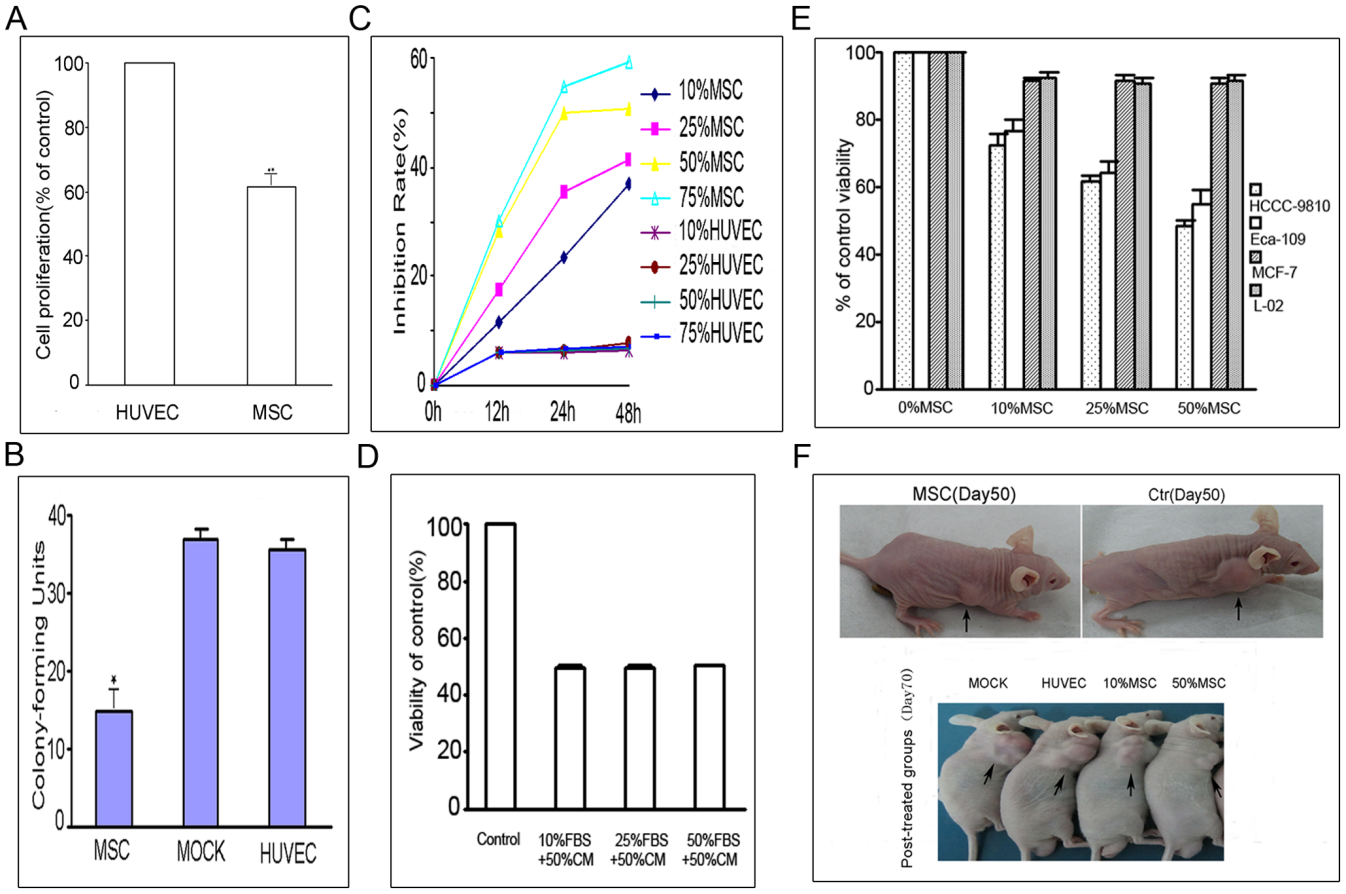
The inhibitory effect of hUC-MSCs on intrahepatic cholangiocarcinoma. HCCC-9810 and HUVEC controls cells were co-cultured with hMSCs for 48 h, after which cells were harvested for counting. The number of cells is represented as the mean±SD of three independent experiments. Results are shown as the percentage of cell number compared with the number of HUVEC control cells. Compared with that of the control, the cell proliferation rate decreased 38.58% (**P<0.05; A). Colony-forming assays showed that there were significantly fewer colony-forming units of HCCC-9810 cells treated with conditioned media from hUC-MSCs than that of control cells. The mean numbers of colony-forming units in the hUC-MSC media treatment group, and the HUVEC media control group were 14.7 and 35.7, respectively (*P*<0.01, B). HCCC-9810 cells were treated with various concentrations of conditioned media from hUC-MSCs (10%,25%,50% and 75%) for 12, 24 and 48 h. MTT assays showed that the inhibitory effects of test groups were significantly higher than that of the HUVEC control (C). The inhibition rate increased to 52.84% in the test group, while it only reached 6.87% in the controls after treatment for 48 h. This growth inhibition was not due to a lack of required nutrients due to consumption during preparation of the conditioned media since cell proliferation did not change when treated with 50% hUC-MSC conditioned media together with increasing concentrations of fetal calf serum (10%, 25%, and 50%) (D). The inhibitory effect was not limited to HCCC-9810 cells (E). There was a similar effect with Eca-109 cells, but not with MCF-7 or L-02 cells. When HCCC-9810 cells were co-injected with hUC-MSCs, smaller tumors formed (arrow) compared to those of HUVEC controls, and the formed tumors shrank in size after injection of conditioned media from hUC-MSC (F).

The numbers of HCCC-9810 cells co-cultured with hMSCs and HUVECs for 48 h were 7.72×10^5^ and 12.57×10^5^, respectively. The hMSC-induced inhibitory effects on tumor cell proliferation were significantly greater than in controls. Compared to that of the control, the cell proliferation rate decreased 38.58% (*P*<0.05; Fig. 2A). In order to determine whether the cultured hUC-MSCs microenvironment could inhibit tumor cell growth, we tested the effect of conditioned media from hUC-MSCs on the ability of HCCC-9810 cells to form colonies. As shown in Fig. 2B, we found that treatment of HCCC-9810 cells with 50% hUC-MSCs conditioned media led to a significant reduction in the number of colony-forming units relative to control cells. The mean numbers of colony-forming units in the hUC-MSC media treatment group, and in the HUVEC media control group were 14.7 and 35.7, respectively (*P*<0.01). We further performed MTT assays to measure the inhibitory effect. To determine whether these results represented a nonspecific response to the conditioned media from hUC-MSCs, we performed similar experiments using infused human umbilical vein endothelial cells (HUVECs), which expressed similar levels of MHC class I and II compared with hMSCs [12]. As shown in Fig. 2C, HCCC-9810 cells treated with hUC-MSC-conditioned media resulted in dose-dependent and time-dependent inhibition of cell proliferation. The proliferation inhibition rate increased from 6.21% to 49.86% when HCCC-9810 cells were cultured with 50% hUC-MSC-conditioned media for 24 h. In order to exclude the possibility that this growth inhibition was due to a lack of required nutrients due to consumption during preparation of the conditioned media, we treated HCCC-9810 cells with 50% hUC-MSC conditioned media together with increasing concentrations of fetal calf serum (10%, 25%, and 50%), and found that the cell proliferation did not change (Fig. 2D).

To further evaluate the effects of MSC cultures on cell proliferation of cells other than HCCC-9810 cells, we treated two different human tumor cell lines, and one normal human cell line with various concentrations MSC conditioned media for 24 h. As can be seen in Fig. 2E, the human esophageal carcinoma cell line Eca-109 was inhibited in a manner similar to that observed for HCCC-9810 cells, whereas the breast cancer tumor line MCF-7, and the human liver cell line L-02 had no such effect.

### Tumor Formation was Inhibited by hUC-MSCs in BALB/c Nude Mice

Results showed that on the 50th day after injection, the mice injected with HCCC-9810 cells and hUC-MSCs had a lower tumor incidence than the control groups, and the mean volume of tumors of the mice injected with tumor cells and MSCs was dramatically lower than that of control groups: 7 mice developed detectable tumors on day 35–40 (average tumor volume = 1.3 cm^3^ on day 50), and 3 mice had not developed any tumors when they were killed on day 50. In contrast, mice injected with HCCC-9810 only, or a mixture of HCCC-9810 and HUVEV, formed detectable tumors (average tumor volumes were 2.6 cm^3^ and 2.5 cm^3^, respectively on day 50) (*P*<0.01, Table 1).

**Table 1:** Tumor transplantation of BALB/c nude mice.

Groups (n = 10)	Subgroups (Day 50–70)	Number of mice with tumor formation	Average Tumor volume (cm^3^)
1.HCCC-9810+MSC		7	1.3**
2.HCCC-9810+HUVEC		10	2.5
3.MSC		0	0
4.HCCC-9810		10	2.6
	4.1+MSC-CM	3	1.4*
	4.2+HUVEC-CM	3	3.5
	4.3No treatment	4	3.6

Both HCCC-9810 and hUC-MSC or HCCC-9810 and HUVEC cells were mixed and subcutaneously inoculated into the nape region of BALB/c nude mice respectively. Tumor volume was calculated (cm^3^) = 1/2×lengt×width^2^. Tumor formation and average volume were significantly different between the test group (HCCC-9810+MSC) and the control groups (**P<0.05, versus controls). For the subgroups with only HCCC-9810 cells, the tumor volume in the post-treated group, which had been injected with conditioned media from hUC-MSC, was significantly lower than that of the control (*P<0.01).

From day 50 to day 70, mice in the three subgroups derived from Group 4 were injected with conditioned media from hUC-MSCs or HUVECs in the tumor sites, or received no treatment. The mean tumor volume in the hUC-MSCs group was significantly smaller compared with that of the control. The average tumor volume decreased to 1.4 cm^3^ in the MSCs group, whereas it continued to increase to 3.5 cm^3^ in the HUVEC control group by day 70 (*P*<0.01, Fig. 2F). Taken together, these observations suggested that hUC-MSCs may inhibit tumor growth in animals.

### The hUC-MSCs Conditioned Media Treatment Resulted in an Induction of Apoptosis in Intrahepatic Cholangiocarcinoma Cells

**Figure 3:**
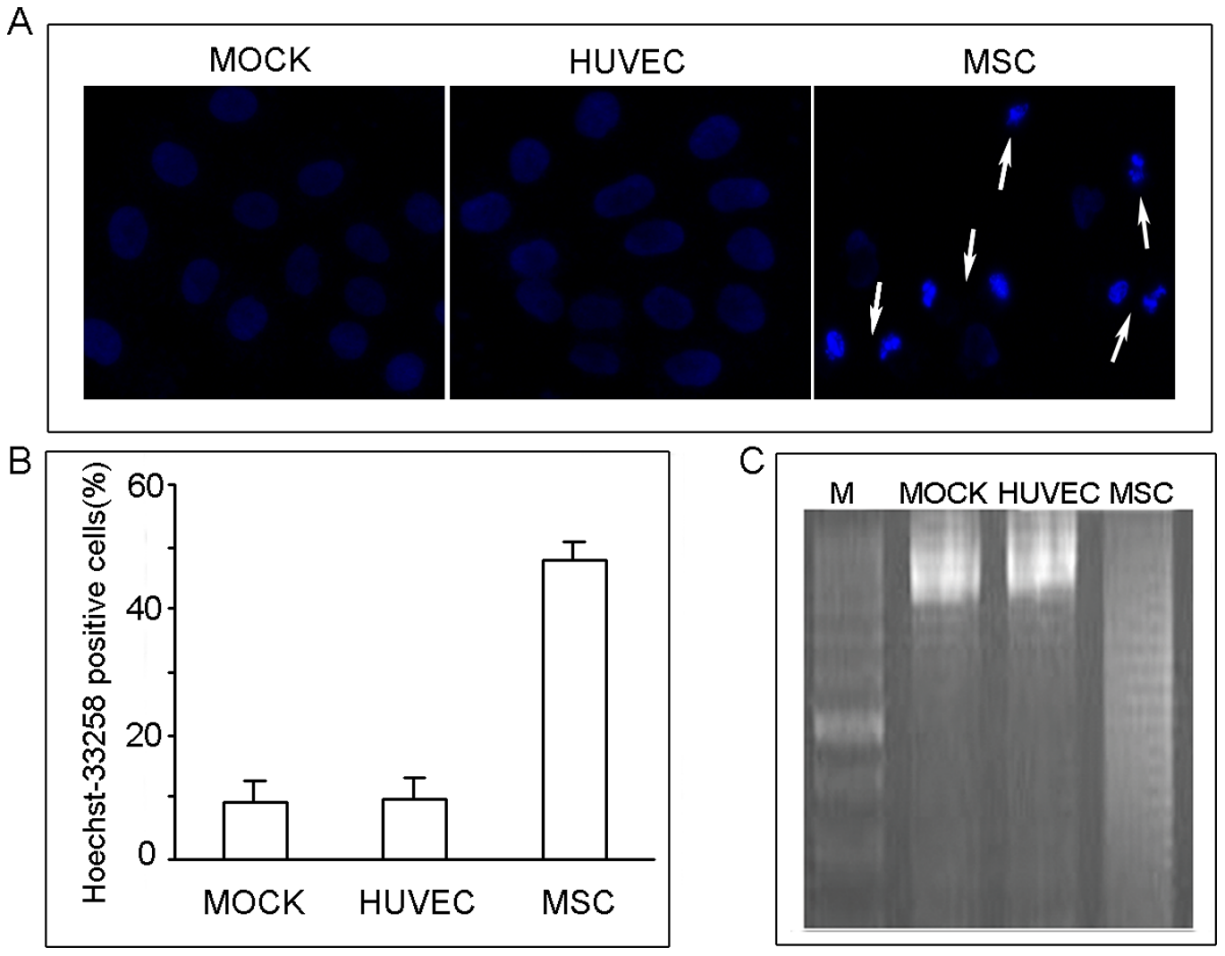
Induction of apoptosis by hUC-MSCs conditioned media in HCCC-9810 cells. Cells were treated with 50% conditioned media from hUC-MSCs or HUVEC or without conditioned media (MOCK) for 48 h. The apoptotic tumor cells stained with Hoechst 33258 are designated by the arrow (A, 400×). The histogram shows the rates of Hoechst 33258-positive cells (*P*<0.05, B). Cells were incubated with 50% hUC-MSC conditioned media for 48 h. DNA fragmentation was detected by 1.5% agarose gel containing ethidium bromide. A representative blot was shown from three independent experiments (C).

As shown in Fig. 3A, treatment of HCCC-9810 cells with 50% conditioned media for 48 h resulted in apoptosis. We counted the apoptotic cells depending on the presence of cell rounding, detachment, and nuclear fragmentation [20]. The means ±SEM for the percentage of apoptotic cells in the treatment group and the control groups were (48.1±2.98) %, (9.3±3.05) %, and (9.6±3.51)%, respectively. Differences between the treatment group and control groups were statistically significant (*P*<0.05, Fig. 3B).

We further examined apoptosis using a DNA ladder assay. After treating the HCCC-9810 cells with 50% conditioned media for 48 h, typical DNA ladders were clearly visible in EtBr-stained gels (Fig. 3C).

### Inhibition of Akt Signaling Using Conditioned Media from hUC-MSC Cultures in Tumor Cells

To understand the mechanism of the inhibition of intrahepatic cholangiocarcinoma cells by hUC-MSC conditioned media, we examined the effects of conditioned media on the PI3K/Akt signaling pathway, which is a critical pathway for intrahepatic cholangiocarcinoma growth and survival. Immunoblot analysis showed that, compared with control groups, treatment of HCCC-9810 cells with hUC-MSC conditioned media resulted in reduced phosphorylation of PI3K^Y458^, PDK1^Ser241^, Akt^Thr308^, and Akt^Ser473^, while the total Akt level did not change. To further assess whether the known target of Akt was also reduced, we measured the expression of phosphorylated GSK-3β. Consistent with the observed reduction in phospho-Akt, the levels of phospho-GSK-3β^Ser9^ in HCCC-9810 cells treated with conditioned media from hUC-MSc was down-regulated. The total GSK-3β remained unchanged.

**Figure 4:**
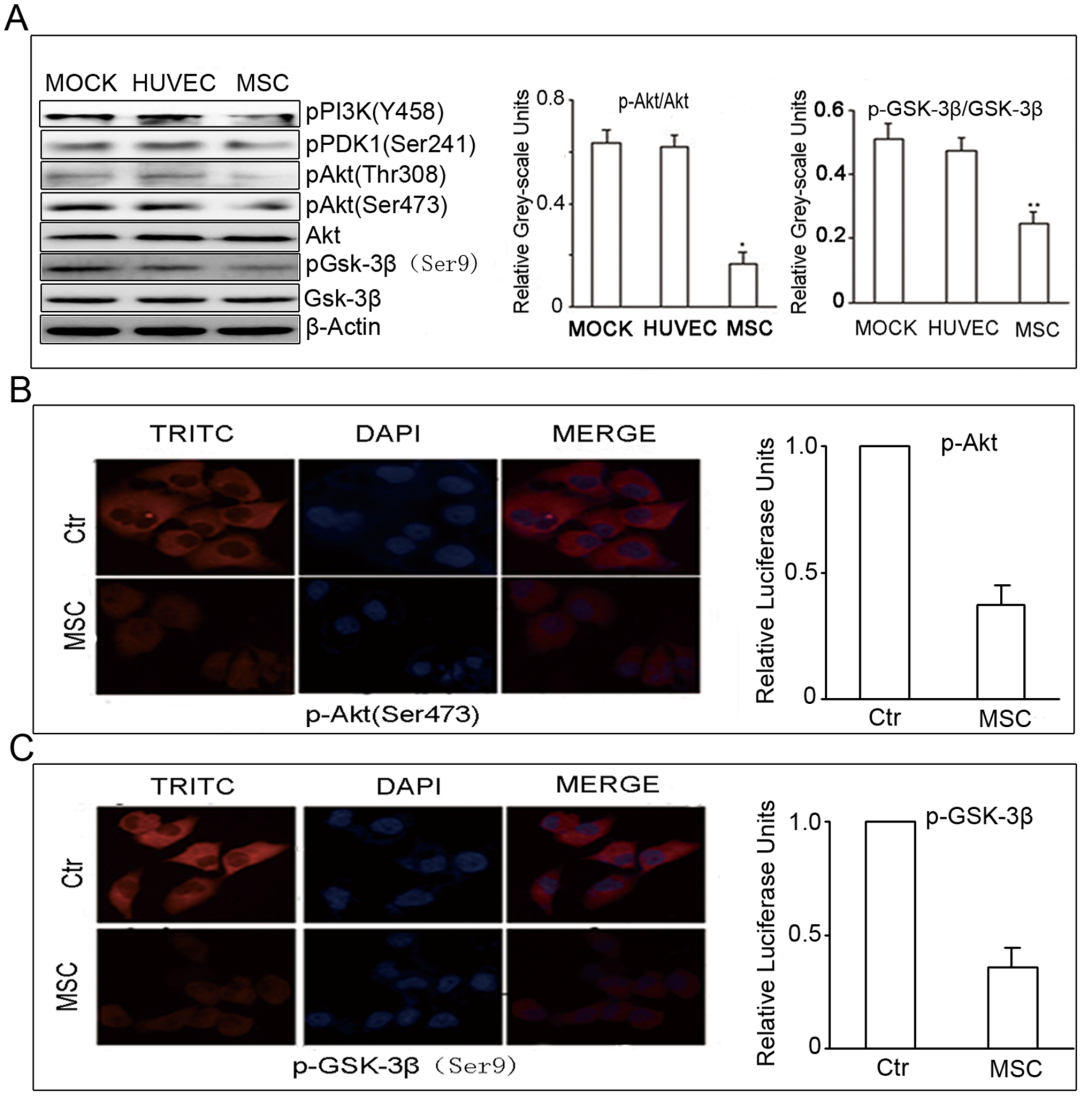
Effect of conditioned media from hUC-MSCs on the Akt signaling pathway. Immunoblot analysis showed that treatment of HCCC-9810 cells with 50% conditioned media from hUC-MSCs cultures led to the down-regulation of p-PI3K, p-PDK1^Ser241^, p-Akt^Thr308^, p-Akt^sSer473^ and p- GSK-3β^Ser9^ expression. However, this did not occur in HCCC-9810 cells with 50% conditioned media from HUVEC cultures. There were substantial differences in the phospho- to total Akt and Gsk-3β ratios between the treated group and control group (A). After treatment for 48 h with 50% conditioned media from hUC-MSCs cultures, the expression of phospho-Akt (B, 200×) and phospho-GSK-3β (C, 200×) in HCCC-9810 cells assessed by immunofluorescence. Results shown are representative of three independent experiments.

However, HUVEC conditioned media failed to down-regulate the expression of these proteins. In the HUVEC control group and MSCs group, the ratios of phospho- to total Akt were 0.65±0.04 and 0.19±0.02, respectively, while the ratios of phospho- to total Gsk-3β was 0.48±0.03 and 0.22±0.05, respectively. There were substantial differences in the phospho- to total Akt and Gsk-3β ratios between the treated group and control group (*P<0.001; **P<0.01, versus controls, respectively, Figure 4A).

Furthermore, we examined the expression of phospho-Akt^Ser473^ and phospho-GSK-3β^Ser9^ through indirect immunofluorescence staining. Compared to the controls, the relative luciferase activities for p-Akt and p-GSK-3β were 37.3±0.8% and 35.7±1.2%, respectively. In contrast to the control, the immunofluorescence staining of phospho-Akt^Ser473^ and phospho-GSK-3β^ser9^ in the treatment group was significantly weaker than that of control (*P*<0.01, p-Akt; *P*<0.01, p-GSK-3β, Figure 4B and 4C).

These effects appeared to be specific because HCCC-9810 cells treated with hUC-MSC cultures did not alter the levels of either phosphorylated ERK1/2 or phosphorylated MEK1/2 in HCCC-9810 cells (data not shown). Together, these findings demonstrate that hUC-MSC cultures specifically inhibited Akt activation within HCCC-9810 cells.

### Down-regulation of Wnt Signaling in Tumor Cells by Conditioned Media from hUC-MSC Cultures

**Figure 5:**
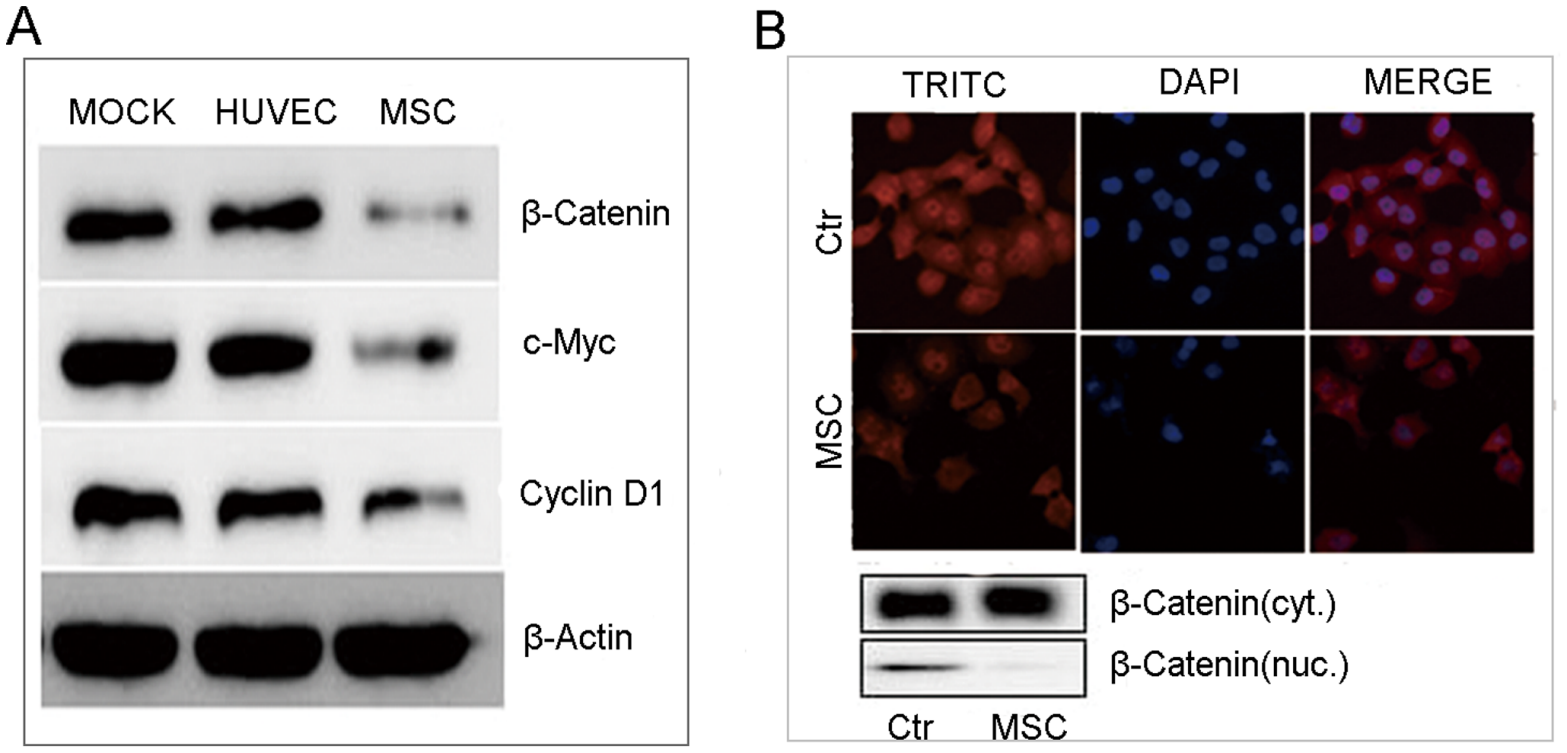
Effect of conditioned media from hUC-MSCs on the Wnt signaling pathway of HCCC-9810 cells. Immunoblot analysis showed that the treatment of HCCC-9810 cells with 50% conditioned media from hUC-MSCs cultures led to the down-regulation of β-catenin, c-Myc, and cyclin-D1 expression, while the levels of these proteins in control groups was unchanged (A). After treatment for 48 h, 50% conditioned media from hUC-MSCs cultures prevented nuclear translocation of β-catenin in HCCC-9810 cells (B). Results shown are representative of three independent experiments.

Since the proliferation of tumor cells can be regulated by canonical Wnt signaling [22], [23], we proposed that this signal transduction pathway might be involved in governing the inhibitory effect on intrahepatic cholangiocarcinoma cells mediated by hMSCs. Our immunoblot results showed that treatment of HCCC-9810 cells with hUC-MSC conditioned media resulted in the down-regulation of β-catenin. To further assess whether known β-catenin targets were also reduced, we analyzed the expression of c-Myc and cyclin-D1. Consistent with the observed reduction in β-catenin, the levels of c-Myc and cyclin-D1 in HCCC-9810 cells receiving hUC-MSCs conditioned media were also down-regulated. However, conditioned media from HUVEC failed to down-regulate the expression of β-catenin, c-Myc or cyclin-D1 (Fig. 5A). Since one of the hallmarks of activated Wnt signaling is the accumulation of nuclear β-catenin [24], we examined the expression and sub-cellular distribution of β-catenin in different treatment groups. Sub-cellular protein fractionation and immunofluorescence cytology showed that treatment of HCCC-9810 cells with conditioned media from hUC-MSC resulted in a decrease in β-catenin nuclear assembly (Fig. 5B). These results are consistent with the hypothesis that soluble molecules in conditioned media released from hUC-MSC cultures might inhibit tumor cell proliferation by the Wnt signaling pathway [14].

### The hUC-MSC Conditioned Media Decreased β-catenin Expression and Inhibited Wnt Signaling by Regulating GSK-3β Activity by the AKT Signaling Pathway

**Figure 6:**
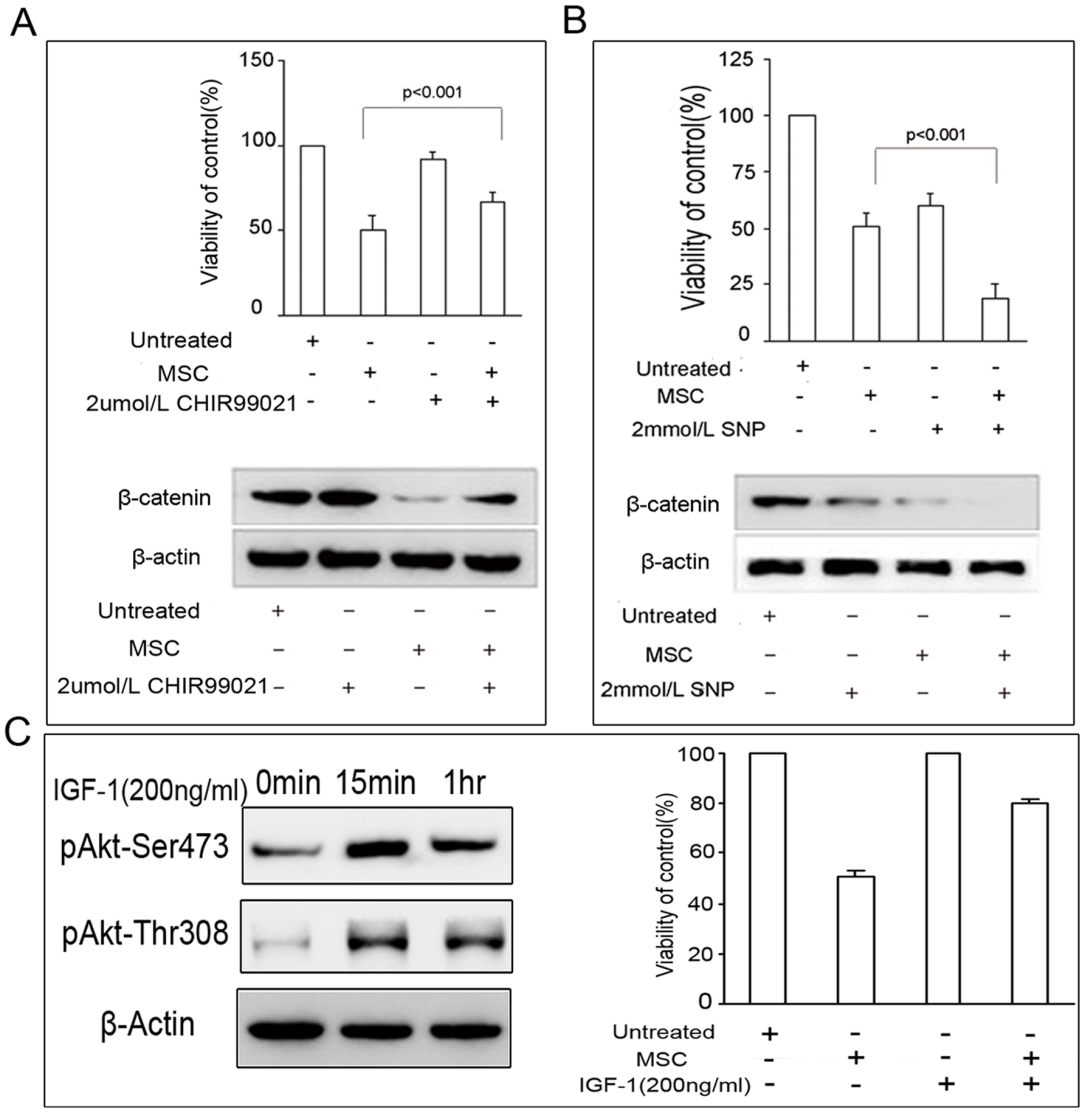
Effect of the GSK-3β inhibitor/activator, and IGF-1 on hUC-MSCs-mediated intrahepatic cholangiocarcinoma cell toxicity. HCCC-9810 cells were incubated with the GSK-3β activator SNP, and the GSK-3β inhibitor CHIR99021 as a single treatment or in combination with hUC-MSC conditioned media for 48 h, and cell survival was measured using the MTT assay (A, B). A similar MTT assay was carried out using IGF-1 (C). The expression of β-catenin and phospho-Akt (Ser 437 and Thr308) was measured by immunoblot analysis.

**Figure 7:**
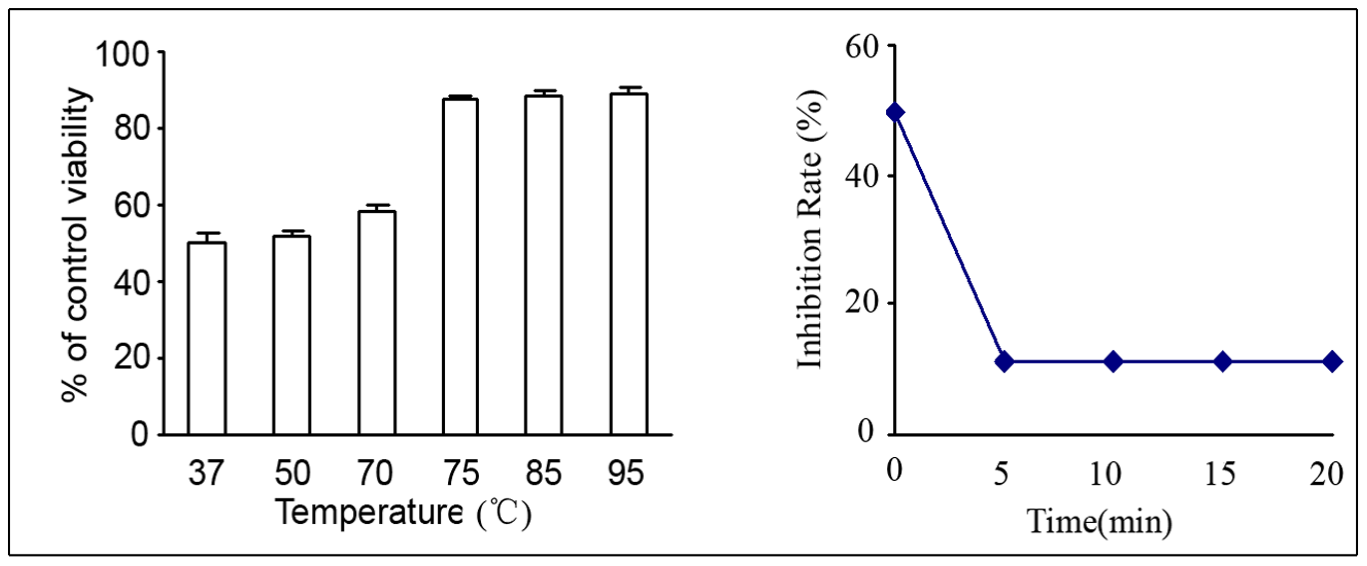
Thermolability of components of conditioned media. The conditioned media were heated to various temperatures and cooled to room temperature prior to preparing the 50% conditioned media. When samples were heated to 75°C or above for 5 mins or longer, the growth inhibition decreased from 49.8% to 11.5%.

It has been shown that activated Akt can effectively suppress the role of GSK-3β in regulating the degradation of β-catenin [25]. To analyze whether GSK-3β was involved in the cross-talk between nuclear translocation of β-catenin and Akt signaling, we treated HCCC-9810 cells with the GSK-3β inhibitor or activator in combination with hUC-MSC conditioned media. As shown in Fig. 6A, the GSK-3β inhibitors CHIR99021 significantly rescued HCCC-9810 cells from the inhibitory effects of hUC-MSCs conditioned media (*P*<0.001), whereas the GSK-3β activator SNP significantly increased the inhibitory effects of hUC-MSCs conditioned media (*P*<0.001). Treatment with CHIR99021 increased β-catenin protein levels in the conditioned media-treated group, while SNP treatment resulted in decreased β-catenin protein levels (Fig. 6B). To further prove that the hUC-MSC conditioned media inhibited Wnt signaling by regulating GSK-3β activity by the Akt signaling pathway, we treated HCCC-9810 cells with IGF-1 in combination with hUC-MSC conditioned media. As shown in Fig. 6C, IGF-1 increased the expression of phospho-Akt (Ser 437 and Thr 308), which meant that IGF-1 activated the Akt signaling pathway. We further treated with hUC-MSC conditioned media for 24 h, with or without IGF-1 (200 ng/mL) preincubation for 15 min. The results showed that exposure to IGF-1 significantly blocked the inhibitory effects of hUC-MSC conditioned media on HCCC-9810 cells (*P*<0.05). In terms of characterization of the active factor(s), the substance(s) passed through a 0.22 µm filter, and was thermo-labile. We heated the conditioned media to various temperatures and cooled them to room temperature prior to preparing the 50% conditioned media. We found that when samples were heated to 75°C or above for 5 mins or longer, the growth inhibition decreased from 49.8% to 11.5% (Fig. 7).

## Discussion

Recent studies have indicated that mesenchymal stem cells exhibit a marked tropism for tumors. However, there is some controversy regarding the mechanisms by which the cells exert effects on tumor progression. Some investigators have suggested that MSCs promote tumor progression and metastasis [26]–[28], while others have reported that MSCs suppress tumor growth [12]–[15]. These discrepancies may be due to differences in tumor models, the heterogeneity of MSCs, the dose or timing of MSCs treated, or other unknown factors [29]. Some of these studies have suggested that extracts from hMSCs might play a role in the growth of certain human tumors. As precursors of stromal cells, MSCs can generate the extra-cellular matrix which can support hematopoiesis within the bone marrow microenvironment [30]. Therefore, it is possible that within the tumor microenvironment, stromal components derived from MSCs may play a role in the development of tumors. In this regard, it has been previously shown that conditioned media from Z_3_ hMSCs could suppress tumor cell proliferation by the secretion of soluble factors that are involved in the Wnt signaling pathway [15]. Our data suggest that extracts from hUC-MSCs cultures may play a role in the progression of intrahepatic cholangiocarcinoma. In the current study, cell proliferation and apoptosis assays demonstrated that hUC-MSC cultures inhibited proliferation and induced apoptosis of HCCC-9810 tumor cells in a dose- and time-dependent manner.

Our next aim was to identify the mechanism by which the observed increase in apoptosis occurred in HCCC-9810 cells. The Wnt/β-catenin pathway plays a major role in intrahepatic cholangiocarcinoma cell growth, tissue homeostasis, and cancer susceptibility [31], [32]. Dysregulation of β-catenin and other Wnt components lead to nuclear localization of β-catenin, activation of Wnt target genes, including c-Myc, cyclin D1, cyclooxygenase-2, matrix metalloproteinase-7, gastrin, and ITF-2 [33]–[39] and enhance tumor formation [40]. This pathway is activated when ligands bind to the cell membrane Wnt receptor, resulting in inhibition of phosphorylation of β-catenin by disrupting a complex consisting of the APC, axin, and GSK-3β proteins [41]. Furthermore, it has been reported that there is an important physiological link between Akt activation and intrahepatic cholangiocarcinoma cell survival [42], [43]. GSK-3β is a key molecule in the PI3K/Akt signaling pathway, and activated Akt has been shown to effectively suppress the role of GSK-3β in regulating the degradation of β-catenin [25]. Based on previous studies, we anticipated that the inhibitory effect of hUC-MSCs on intrahepatic cholangiocarcinoma cells may be mediated by both the Wnt and Akt signaling pathway.

We, therefore, investigated the effects of hUC-MSC cultures on HCCC-9810 cells at the molecular level. It has been shown that in response to certain growth stimuli, PI3K-activated AKT can phosphorylate GSK-3β at Ser^9^, leading to inactivation of GSK-3β and augmentation of β-catenin-TCF4 transcriptional activity [44]–[47]. Our data suggest that cultures from hUC-MSCs may act in a similar manner. We found that conditioned media from hUC-MSCs can affect PI3K/Akt signal transduction through inhibition of PDK1 phosphorylation and inhibit Akt activity resulting in activation of GSK-3β. This may lead to inhibition of nuclear β-catenin translocation, which down-regulates the expression of target genes c-Myc and cyclin D1, and induces the apoptosis of HCCC-9810 cells.

Inhibition of nuclear β-catenin translocation is likely to be due to the observed inhibition of Akt activity by hUC-MSC conditioned media. This, in turn, could have triggered activation of GSK-3β function because activated Akt has been shown to effectively suppress GSK-3β levels which regulate the degradation of β-catenin [48]. This is supported by the findings that the GSK-3β activator SNP significantly increased the cytotoxic effects of hUC-MSCs conditioned media and decreased the expression of β-catenin, whereas the GSK-3β inhibitors CHIR99021 inhibited hUC-MSCs conditioned media -mediated HCCC-9810 cell death and increased β-catenin protein expression. We treated HCCC-9810 cells with IGF-1 in combination with hUC-MSC conditioned media, and found an increase in the expression of phosphor-Akt (Ser 437 and Thr308). IGF-1 significantly blocked the inhibitory effects of hUC-MSCs conditioned media on HCCC-9810 cells. However, the direct inhibitory target of hMSCs on HCCC-9810 cells has not been investigated in the current study.

**Figure 8:**
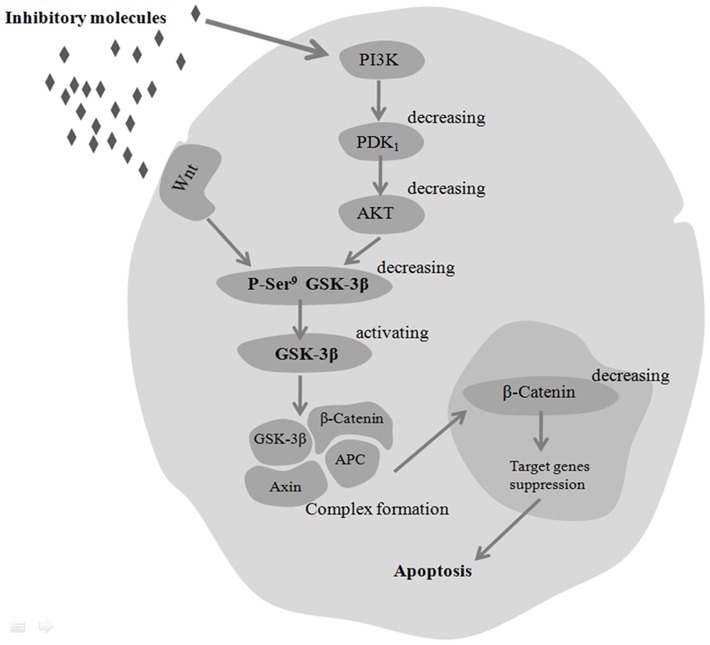
Proposed model by which hUC-MSC cultures mediates cross-talk between Wnt and Akt signaling in HCCC-9810 cells. Inhibitory molecules in hUC-MSC conditioned media mediate Akt phosphorylation in HCCC-9810 cells, thereby decreasing phosphorylation of GSK-3βand increasing its activity. Activation of GSK-3βleads to decreased cellularβ-catenin levels. A decrease in β-catenin translocation to the nucleus to bind TCF4, and decreases the transcription of its specific target genes resulting in apoptosis.

Based on our findings, we propose a schematic model of how the hUC-MSC cultures could have affected various molecular events by the cross-talk between the PI3K/Akt and the Wnt/β-catenin signaling pathways, leading to apoptosis in human HCCC-9810 cells (Fig. 8). In this scheme, inhibitory molecules in hUC-MSC conditioned media mediate Akt phosphorylation in HCCC-9810 cells, thereby decreasing phosphorylation of GSK-3β and increasing its activity. Activation of GSK-3βleads to increased degradation of β-catenin, and decreased cellular β-catenin levels. A reduction in β-catenin translocation to the nucleus to bind TCF4, leads to decreased transcription of its specific target genes resulting in apoptosis.

Taken together, our data show that hUC-MSCs cultures can inhibit the proliferation and induce apoptosis of human intrahepatic cholangiocarcinoma cells. The molecular cross-talk between the PI3K/Akt and the Wnt/β-catenin signaling pathways is involved in this inhibitory effect, with GSK-3β as the key enzyme bridging these pathways. We speculate that the hUC-MSC microenvironment might have an essential role in inducing apoptosis in tumor cells by inhibiting the PI3K/Akt and the Wnt/β-catenin signaling pathways. A more detailed understanding of the cross-talk role between the Akt and Wnt signaling pathway may be helpful in the investigation of the inhibitory molecules present in hMSC extracts, and possibly improve therapeutic strategies involving hMSC-mediated targeting of tumor cells.
