# Correction: Suppression of Cholangiocarcinoma Cell Growth by Human Umbilical Cord Mesenchymal Stem Cells: A Possible Role of Wnt and Akt Signaling

**DOI:** 10.1371/annotation/7fff93fa-8e80-41f3-b35a-e658cb7256a9

**Published:** 2013-07-02

**Authors:** Juan Liu, Guoqing Han, Hui Liu, Chengyong Qin

There is a grant number missing from the Funding Statement. The following is the missing information:

This work was supported by Grant No. ZR2010HM046 from the Shandong Science and Technology Committee of China.

